# Supplementary material for: Comparing Different Sticky Traps to Monitor the Occurrence of Philaenus spumarius and Neophilaenus campestris, Vectors of Xylella fastidiosa, in Different Crops
Source: Insects. 2023 Sep 21;14(9):777. doi: 10.3390/insects14090777 (PMC10531974; doi:10.3390/insects14090777)
Supplement: Supplementary file 1 [file insects-14-00777-s001.zip › Supplement tables.pdf]

Supplementary Table S1. Repeated measures ANOVA for the captures of spittlebugs and others Auchenorrhyncha species on colored traps hanged on olive trees

Site A - 2018													
Species	Traps	May		June		July		August		September		Mean months	
P. spumarius	Yellow	-		3.70	c	2.77	c	6.68	b	0.80	b	3.49	c
	Y-circle	-		5.68	c	3.37	c	6.68	b	1.32	b	4.26	c
	Y-Line	-		5.20	c	4.03	c	8.87	b	1.47	b	4.89	c
N. campestris	Yellow	-		0.58	c	0.17	c	1.75	b	0.30	b	0.70	c
	Y-circle	-		0.50	c	0.27	c	0.92	b	0.17	b	0.46	c
	Y-Line	-		0.48	c	0.13	c	2.02	b	0.52	b	0.79	c
Others Auchenorrhyncha species	Yellow	-		32.78	b	20.60	b	55.23	a	24.00	a	33.15	b
	Y-circle	-		46.25	a	27.53	ab	58.22	a	24.90	a	39.23	ab
	Y-Line	-		43.98	ab	35.96	a	59.92	a	25.58	a	41.36	a
F-value				50.37		44.80		51.87		35.56		102.7	
P-value				< 0.00001		< 0.00001		< 0.00001		< 0.00001		< 0.00001	
				***		***		***		***		***	

Site A – 2019													
Species	Traps	May		June		July		August		September		Mean months	
P. spumarius	Yellow	0.75	b	4.33	b	1.17	b	1.25	c	0.50	b	1.60	b
	Y-circle	1.13	b	4.08	b	1.50	b	1.50	c	0.46	b	1.73	b
	Y-Line	0.92	b	4.08	b	1.54	b	1.33	c	0.75	b	1.72	b
N. campestris	Yellow	0.17	b	0.33	b	0.25	b	0.04	c	0.00	b	0.16	b
	Y-circle	0.54	b	0.33	b	0.46	b	0.13	c	0.00	b	0.29	b
	Y-Line	0.13	b	0.42	b	0.25	b	0.17	c	0.00	b	0.19	b
Others Auchenorrhyncha species	Yellow	25.63	a	249.88	a	61.67	a	87.58	a	32.25	a	91.40	a
	Y-circle	20.80	a	205.58	a	58.88	a	71.38	b	28.17	a	76.96	a
	Y-Line	20.63	a	255.08	a	63.17	a	72.75	ab	32.17	a	88.76	a
F-value		24.60		51.03		87.03		116.21		34.00		110.75	
P-value		< 0.00001		< 0.00001		< 0.00001		< 0.00001		< 0.00001		< 0.00001	
		***		***		***		***		***		***	

Site B – 2019													
Species	Traps	May		June		July		August		September		Mean months	
P. spumarius	Yellow	0.13	b	1.88	b	2.63	bc	1.88	b	3.38	bc	1.98	b
	Y-circle	0.00	b	1.13	b	3.00	bc	1.63	b	3.63	bc	1.88	b
	Y-Line	0.00	b	1.13	b	2.50	bc	2.88	b	3.50	bc	2.00	b
N. campestris	Yellow	0.00	b	0.25	b	0.00	c	0.00	b	0.00	c	0.05	b
	Y-circle	0.00	b	0.25	b	0.00	c	0.00	b	0.00	c	0.05	b
	Y-Line	0.00	b	0.25	b	0.25	c	0.00	b	0.00	c	0.10	b
Others Auchenorrhyncha species	Yellow	3.38	ab	26.75	a	6.13	abc	20.25	a	12.63	a	13.83	a
	Y-circle	4.00	ab	24.38	a	11.38	a	15.63	a	12.13	a	13.50	a
	Y-Line	7.25	a	30.50	a	9.50	ab	15.50	a	10.25	ab	14.60	a
F-value		5,23		29.49		5.29		23.71		8.87		38.63	
P-value		< 0,00001		< 0.00001		< 0.00001		< 0.00001		< 0.00001		< 0.00001	
		***		***		***		***		***		***	

Supplementary Table S2. Repeated measures ANOVA for the captures of spittlebugs and others Auchenorrhyncha species on colored traps hanged on almond trees

Site A - 2018													
Species	Traps	May		June		July		August		September		Mean months	
P. spumarius	Yellow	-		13.05	a	12.28	ab	53.84	b	12.28	a	22.86	b
	Y-circle	-		14.30	a	11.47	abc	65.82	a	13.32	a	26.23	ab
	Y-Line	-		14.82	a	16.30	a	62.53	ab	13.62	a	26.82	a
N. campestris	Yellow	-		2.17	b	1.72	c	8.64	c	1.58	b	3.53	d
	Y-circle	-		1.57	b	1.87	c	10.16	c	1.70	b	3.82	d
	Y-Line	-		2.60	b	2.77	c	10.57	c	2.05	b	4.50	d
Others Auchenorrhyncha species	Yellow	-		14.97	a	10.23	abc	1.66	c	0.87	b	6.93	c
	Y-circle	-		14.03	a	4.40	cd	1.44	c	1.47	b	5.33	c
	Y-Line	-		17.70	a	7.35	bcd	2.22	c	1.42	b	7.17	c
F-value				18.03		9.74		147.07		45.19		171.8	
P-value				< 0.00001		< 0.00001		< 0.00001		< 0.00001		< 0.00001	
		–		***		***		***		***		***	

Site A – 2019													
Species	Traps	May		June		July		August		September		Mean months	
P. spumarius	Yellow	2.79	bcd	8.13	c	217.00	b	3.21	b	2.54	ab	46.73	c
	Y-circle	3.00	bcd	7.50	c	2.67	b	2.33	b	1.92	ab	3.48	c
	Y-Line	2.92	bcd	6.92	c	2.55	b	1.92	b	2.21	ab	3.30	c
N. campestris	Yellow	1.50	cd	1.63	c	0.38	b	0.42	b	0.29	b	0.84	d
	Y-circle	0.96	d	1.21	c	0.58	b	0.38	b	0.29	b	0.68	d
	Y-Line	1.04	cd	1.04	c	0.42	b	0.33	b	0.21	b	0.61	d
Others Auchenorrhyncha species	Yellow	3.29	bc	21.96	b	7.46	a	8.71	a	4.38	a	9.16	b
	Y-circle	4.88	ab	25.83	ab	6.50	a	7.83	a	4.29	a	9.87	ab
	Y-Line	6.00	a	34.04	a	7.25	a	8.04	a	3.58	a	11.78	a
F-value		10.65		25.92		25.65		28.40		7.62		70.89	
P-value		< 0.00001		< 0.00001		< 0.00001		< 0.00001		< 0.00001		< 0.00002	
		***		***		***		***		***		***	

Site B – 2019													
Species	Traps	May		June		July		August		September		Mean months	
P. spumarius	Yellow	0.13	b	1.50	b	0.88	c	0.88	b	1.38	bc	0.95	b
	Y-circle	0.13	b	2.00	b	1.75	c	0.75	b	1.75	bc	1.28	b
	Y-Line	0.00	b	1.75	b	1.13	c	1.38	b	2.13	abc	1.28	b
N. campestris	Yellow	0.00	b	0.13	b	0.25	c	0.13	b	0.00	c	0.10	b
	Y-circle	0.13	b	0.13	b	0.00	c	0.00	b	0.00	c	0.05	b
	Y-Line	0.00	b	0.00	b	0.13	c	0.00	b	0.00	c	0.03	b
Others Auchenorrhyncha species	Yellow	3.00	a	17.63	a	2.50	bc	6.75	a	3.13	ab	6.60	a
	Y-circle	4.00	a	17.75	a	5.25	a	6.63	a	4.00	ab	7.53	a
	Y-Line	4.63	a	23.38	a	4.50	ab	7.25	a	4.63	a	8.88	a
F-value		3.39		14.21		10.55		14.00		9.18		21.54	
P-value		< 0.0030		< 0.00001		< 0.00001		< 0.00001		< 0.00001		< 0.00001	
		**		***		***		***		***		***	

Supplementary Table S3. Repeated measures ANOVA for the captures of spittlebugs and others Auchenorrhyncha species on colored traps hanged on cherry trees

Site A - 2019													
Species	Traps	May		June		July		August		September		Mean months	
P. spumarius	Yellow	0.71	bc	1.63	b	0.92	abc	1.42	ab	0.83	ab	1.10	c
	Y-circle	0.75	bc	1.50	b	1.21	abc	1.00	ab	0.75	ab	1.04	c
	Y-Line	0.83	abc	3.33	b	0.79	bc	1.08	ab	1.13	a	1.43	c
N. campestris	Yellow	0.08	c	0.25	b	0.29	c	0.04	b	0.00	b	0.13	c
	Y-circle	0.04	c	0.58	b	0.00	c	0.21	b	0.00	b	0.17	c
	Y-Line	0.21	c	0.96	b	0.21	c	0.13	b	0.04	b	0.31	c
Others Auchenorrhyncha species	Yellow	2.29	ab	9.75	a	4.00	a	3.00	ab	1.00	a	4.01	a
	Y-circle	1.33	abc	2.25	ab	2.67	ab	1.88	ab	0.71	a	1.77	bc
	Y-Line	2.58	a	5.63	ab	4.04	a	3.71	a	1.13	a	3.42	ab
F-value		5.42		4.37		4.82		2.63		3.48		8.35	
P-value		< 0.00001		< 0.00001		< 0.00001		0.01		0.00		< 0.00001	
		***		***		***		**		***		***	

Site B – 2019													
Species													
Species	Traps	May		June		July		August		September		Mean months	
P. spumarius	Yellow	0.25	c	2.25	bc	0.75	b	1.13	cd	1.75	ab	1.23	b
	Y-circle	0.13	c	1.13	bc	2.88	ab	1.50	cd	2.38	ab	1.60	b
	Y-Line	0.00	c	2.38	bc	1.88	b	1.00	cd	1.75	ab	1.40	b
N. campestris	Yellow	0.00	c	0.25	c	0.38	b	0.13	d	0.00	b	0.15	b
	Y-circle	0.00	c	0.00	c	0.00	b	0.25	d	0.00	b	0.05	b
	Y-Line	0.00	c	0.13	c	0.13	b	0.25	d	0.00	b	0.10	b
Others Auchenorrhyncha species	Yellow	1.88	a	10.13	abc	4.50	ab	4.38	bc	6.75	a	5.53	a
	Y-circle	1.25	b	11.50	ab	9.63	a	7.38	ab	6.13	a	7.18	a
	Y-Line	0.75	b	16.00	a	9.38	a	10.00	a	5.88	a	8.40	a
F-value		2.21		6.75		6.28		15.34		4.84		18.46	
P-value		0.04		< 0.00001		< 0.00001		< 0.00001		< 0.00001		< 0.00001	
		*		***		***		***		***		***	

Supplementary Table S4. Mean captures ±SE of *Philaenus spumarius* adults by sweep net and on sticky traps at site A and B on olive trees during 2018 and 2019.

		Site A				Site B			
		Mean (± SE)				Mean (± SE)			
Period inspections		Sweep net	Traps	t-values	p-values	Sweep net	Traps	t-values	p-values
2018									
Aug	2nd	4.00 (±0.58)	2.05 (±0.44)	2.69	0.0052	-	-	-	-
Sept	1st	0.25 (±0.10)	0.80 (±0.28)	1.87	0.0348	-	-	-	-
	2nd	1.80 (±0.35)	0.65 (±0.15)	3.00	0.0024	-	-	-	-
Oct	1st	0.15 (±0.08)	0.75 (±0.18)	3.09	0.0019	-	-	-	-
	2nd	0.00 (±0.00)	0.08 (±0.04)	2.13	0.0974	-	-	-	-
2019									
May	1st	0.00 (±0.00)	0.00 (±0.00)	0.00	0.5000	0.00 (±0.00)	0.00 (±0.00)	0.00	0.5000
	2nd	0.65 (±0.20)	0.10 (±0.07)	2.65	0.0058	0.15 (±0.08)	0.04 (±0.04)	1.24	0.1113
June	1st	1.90 (±0.48)	1.40 (±0.34)	0.85	0.1995	4.00 (±0.17)	0.04 (±0.04)	2.24	0.0153
	2nd	0.90 (±0.24)	2.10 (±0.38)	2.69	0.0053	0.50 (±0.14)	1.33 (±0.27)	2.62	0.0061
July	1st	0.60 (±0.21)	0.90 (±0.25)	0.92	0.1823	0.65 (±0.17)	1.63 (±0.27)	2.89	0.0304
	2nd	0.95 (±0.25)	0.90 (±0.23)	0.15	0.4411	0.30 (±0.13)	1.08 (±0.34)	2.00	0.0258
Aug	1st	1.00 (±0.23)	0.75 (±0.26)	0.72	0.2378	0.50 (±0.18)	0.63 (±0.20)	0.45	0.3258
	2nd	0.30 (±0.11)	1.25 (±0.27)	3.28	0.0011	0.20 (±0.09)	1.50 (±0.37)	3.13	0.0016
Sept	1st	0.10 (±0.07)	0.60 (±0.34)	1.46	0.0763	0.30 (±0.13)	3.96 (±0.54)	5.35	0.0000
	2nd	0.25 (±0.10)	1.05 (±0.42)	1.85	0.0357	0.60 (±0.17)	0.25 (±0.17)	1.43	0.0796
Oct	1st	0.10 (±0.07)	0.10 (±0.10)	0.00	0.5000	0.35 (±0.13)	0.46 (±0.17)	0.49	0.3138
	2nd	0.05 (±0.05)	0.50 (±0.22)	1.96	0.0284	0.05 (±0.05)	0.42 (±0.13)	2.39	0.0107

Supplementary Table S5. Mean captures ±SE of *Philaenus spumarius* adults by sweep net and on sticky traps at ~~in~~ site A and B on almond trees during 2018 and 2019.

		Site A				Site B			
**Period inspections**		**Mean (± SE)**		**t-values**	**p-values**	**Mean (± SE)**		**t-values**	**p-values**
		**Sweep net**	**Traps**			**Sweep net**	**Traps**		
**2018**									
Aug	2nd	1.15 (±0.24)	27.15 (±1.11)	22.96	<0,00001	-	-	-	-
	1st	0.95 (±0.25)	12.25 (±1.57)	7.10	<0,00001	-	-	-	-
Sept	2nd	0.90 (±0.27)	4.74 (±0.38)	8.45	<0,00001	-	-	-	-
	1st	0.15 (±0.08)	2.55 (±0.92)	2.60	0.0066	-	-	-	-
Oct	2nd	0.20 (±0.09)	0.10 (±0.04)	0.87	0.1944	-	-	-	-
**2019**									
	1st	0.00 (±0.00)	0.00 (±0.00)	0.00	0.5000	0.05 (±0.05)	0.00 (±0.00)	1.00	0.1618
May	2nd	0.15 (±0.08)	0.20 (±0.12)	0.35	0.3641	0.15 (±0.08)	0.08 (±0.06)	0.68	0.2496
	1st	1.55 (±0.53)	3.30 (±0.51)	2.38	0.0112	0.35 (±0.17)	0.21 (±0.08)	0.80	0.2152
June	2nd	2.10 (±0.41)	3.00 (±0.44)	1.49	0.0716	0.15 (±0.08)	1.54 (±0.30)	4.11	0.0001
	1st	0.75 (±0.22)	1.60 (±0.32)	2.20	0.0169	0.15 (±0.11)	0.46 (±0.12)	1.87	0.0345
July	2nd	0.40 (±0.13)	0.85 (±0.25)	1.57	0.0672	0.30 (±0.13)	0.96 (±0.26)	1.58	0.0604
	1st	0.50 (±0.18)	1.10 (±0.27)	1.83	0.0375	0.00 (±0.00)	0.21 (±0.08)	2.24	0.0152
Aug	2nd	0.15 (±0.08)	1.35 (±0.33)	3.48	0.0006	0.05 (±0.05)	0.79 (±0.19)	3.47	0.0007
	1st	0.10 (±0.07)	2.45 (±0.55)	4.20	0.0001	0.00 (±0.00)	1.75 (±0.28)	5.75	<0,00001
Sept	2nd	0.05 (±0.05)	1.10 (±0.43)	2.43	0.0099	0.00 (±0.00)	0.13 (±0.07)	1.65	0.0532
	1st	0.00 (±0.00)	0.45 (±0.18)	2.44	0.0098	0.00 (±0.00)	0.50 (±0.17)	2.68	0.0053
Oct	2nde	0.00 (±0.00)	1.55 (±0.28)	5.62	0.0000	0.00 (±0.00)	1.17 (±0.32)	3.30	0.0099

Supplementary Table S6. Mean captures ±SE of *Philaenus spumarius* adults by sweep net and on sticky traps at ~~in~~ site A and B on cherry trees during 2018 and 2019.

		**Site A**				**Site B**			
		**Mean (± SE)**				**Mean (± SE)**			
**Period inspections**		**Sweep net**	**Traps**	**t-values**	**p-values**	**Sweep net**	**Traps**	**t-values**	**p-values**
**2019**									
	1st	0.20 (±0.09)	0.20 (±0.00)	2.18	0.0018	0.05 (±0.05)	0.00 (±0.00)	1.10	0.1392
May	2nd	2.15 (±0.34)	0.10 (±0.07)	5.87	0.00001	0.30 (±0.16)	0.13 (±0.07)	0.98	0.1577
	1st	1.50 (±0.27)	0.80 (±0.30)	1.76	0.0433	0.60 (±0.21)	0.33 (±0.13)	1.12	0.1353
June	2nd	2.05 (±0.36)	1.05 (±0.48)	1.66	0.0526	0.25 (±0.10)	1.58 (±0.36)	3.28	0.0010
	1st	1.10 (±0.28)	0.65 (±0.22)	1.26	0.1073	0.50 (±0.20)	1.00 (±0.25)	1.53	0.0669
July	2nd	1.10 (±0.26)	0.35 (±0.17)	2.42	0.01	0.05 (±0.05)	0.83 (±0.21)	3.27	0.0107
	1st	0.80 (±0.20)	0.40 (±0.24)	1.26	0.1068	0.10 (±0.07)	0.29 (±0.09)	1.58	0.0619
Aug	2nd	1.50 (±0.34)	1.10 (±0.35)	0.81	0.2117	0.00 (±0.00)	0.92 (±0.22)	3.86	0.0002
	1st	2.45 (±0.63)	1.00 (±0.44)	1.89	0.033	0.35 (±0.18)	1.95 (±0.37)	3.68	0,000329
Sept	2nd	1.15 (±0.37)	1.00 (±0.36)	0.29	0.3861	0.25 (±0.10)	0.25 (±0.18)	0.00	0.5000
	1st	1.25 (±0.25)	0.85 (±0.38)	0.88	0.1919	0.15 (±0.08)	0.92 (±0.22)	2.98	0.0024
Oct	2nd	0.30 (±0.21)	1.00 (±0.37)	1.65	0.0534	0.60 (±0.17)	1.42 (±0.33)	2.08	0.0218
