# Supplementary figures and images for: Comparing Different Sticky Traps to Monitor the Occurrence of Philaenus spumarius and Neophilaenus campestris, Vectors of Xylella fastidiosa, in Different Crops
Source: Insects. 2023 Sep 21;14(9):777. doi: 10.3390/insects14090777 (PMC10531974; doi:10.3390/insects14090777)

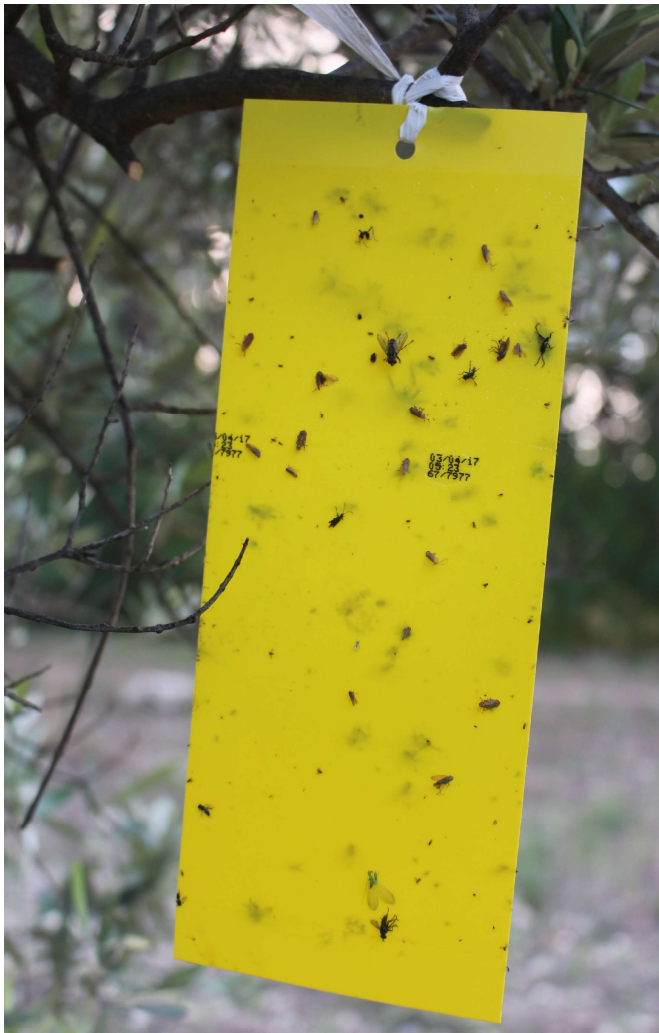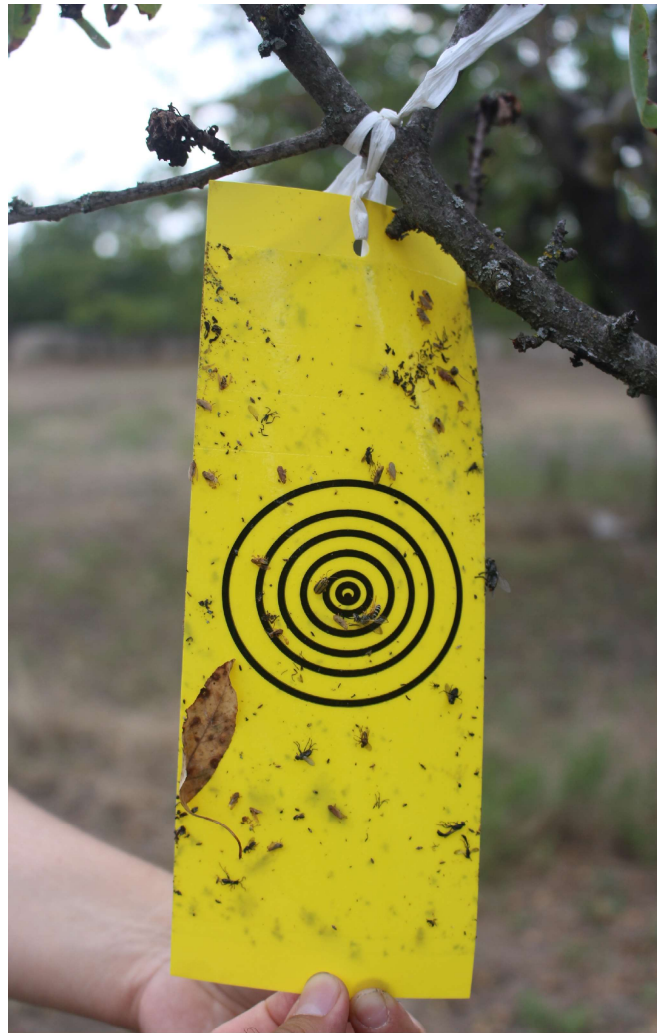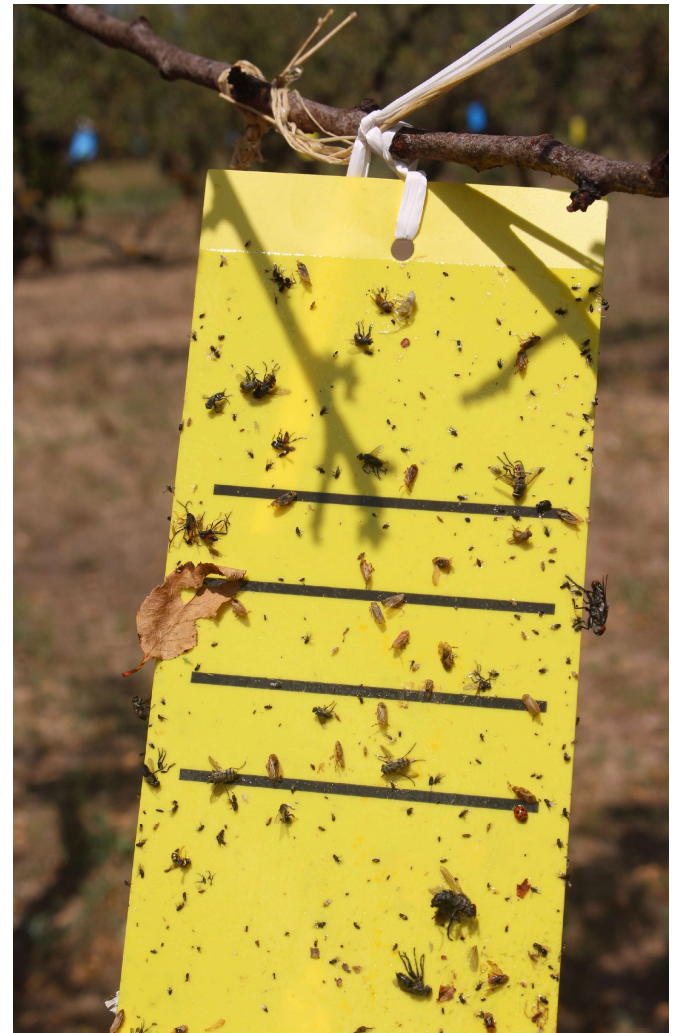

Figure S1. Yellow Sticky traps used in the experiments.

Supplement: Supplementary file 1 [file insects-14-00777-s001.zip › Figure S1.pdf]
